# Supplementary material for: Isoliquiritigenin Derivatives Inhibit RANKL-Induced Osteoclastogenesis by Regulating p38 and NF-κB Activation in RAW 264.7 Cells
Source: Molecules. 2020 Aug 27;25(17):3908. doi: 10.3390/molecules25173908 (PMC7504656; doi:10.3390/molecules25173908)
Supplement: Supplementary file 1 [file molecules-25-03908-s001.pdf]

Isoliquiritigenin derivatives inhibit RANKL-induced osteoclastogenesis by regulating p38 and NF-κB activation in RAW 264.7 cells

Seongtae Jeong [1,#], Seahyoung Lee[2,#], Kundo Kim[3], Yunmi Lee[4], Jiyun Lee[5], Sena Oh[6], Jung-Won Choi[7], Sang Woo Kim[8], Ki-Chul Hwang[9,*] and Soyeon Lim[10,*]

[1] Institute for Bio-Medical Convergence, College of Medicine, Catholic Kwandong University, Gangneung-si, Gangwon-do 210-701, Korea ; 91seongtae@gmail.com

[2] Institute for Bio-Medical Convergence, College of Medicine, Catholic Kwandong University, Gangneung-si, Gangwon-do 210-701, Korea ; sam1017@ish.ac.kr

[3] Department of Chemistry, Kwangwoon University, Seoul 01897, Republic of Korea; vmczl_@naver.com

[4] Department of Chemistry, Kwangwoon University, Seoul 01897, Republic of Korea; ymlee@kw.ac.kr

[5] Institute for Bio-Medical Convergence, College of Medicine, Catholic Kwandong University, Gangneung-si, Gangwon-do 210-701, Korea ; jylee12334@gmail.com

[6] Institute for Bio-Medical Convergence, College of Medicine, Catholic Kwandong University, Gangneung-si, Gangwon-do 210-701, Korea ; bole1305@naver.com

[7] Institute for Bio-Medical Convergence, College of Medicine, Catholic Kwandong University, Gangneung-si, Gangwon-do 210-701, Korea ; gardinia@ hanmail.net

[8] Institute for Bio-Medical Convergence, College of Medicine, Catholic Kwandong University, Gangneung-si, Gangwon-do 210-701, Korea ; doctor7408@gmail.com

[9] Institute for Bio-Medical Convergence, College of Medicine, Catholic Kwandong University, Gangneung-si, Gangwon-do 210-701, Korea ; kchwang@cku.ac.kr

[10] Institute for Bio-Medical Convergence, College of Medicine, Catholic Kwandong University, Gangneung-si, Gangwon-do 210-701, Korea ; redclover77@hanmail.net

# Seongtae Jeong and Seahyoung Lee contributed equally to the work.

* Correspondence: kchwang@cku.ac.kr; Tel.: +82-32-290-2774 (K-C.H.), redclover77@hanmail.net; Tel.: +82-32-290-2777 (S.L.)

Supporting information

Materials and Methods

General Information. ^{1}H NMR spectra were recorded a JEOL JNM-AL400 (400 MHz) spectrometer. Chemical shifts are reported in ppm from tetramethylsilane, with the solvent resonance as the internal standard ($CDCl_3$: δ 7.27 ppm, DMSO-d_6: δ 2.50 ppm). Data are reported as follows: chemical shift, multiplicity (s = singlet, d = doublet, t = triplet, q = quartet, quint = quintet, m = multiplet), coupling constants (Hz), and integration. ^{13}C NMR spectra were recorded on a JEOL JNM-AL400 (100 MHz) spectrometer with complete proton decoupling. Chemical shifts are reported in ppm from tetramethylsilane with the solvent resonance as the internal standard ($CDCl_3$: δ 77.00 ppm, DMSO-d_6: δ 39.51 ppm). Isoliquiritigenin and its 12 derivatives were synthesized as shown in Scheme 1 (compound 1-13) and spectra data match described.[1]

(1) (a) Ren, B.-z.; Ablise, M.; Yang, X.-c.; Liao, B.-e.; Yang, Z. *Med. Chem. Res.* **2017**, *26*, 1871. (b) Zhen, X.-H.; Quan, Y.-C.; Peng, Z.; Han, Y.; Zheng, Z.-J.; Guan, L.-P. *Chem. Biol. Drug Des.* **2016**, *87*, 858. (c) Neves, M. P.; Cravo, S.; Lima, R. T.; Vasconcelos, M. H.; Nascimento, M. S. J.; Silva, A. M. S.; Pinto, M. Cidade, H.; Corrêa, A. G. *Bioorg. Med. Chem.* **2012**, *20*, 25. (d) Jajyapal, M. R.; Sreedhar, N. Y. *Asian J. Pharm. Clin. Res.* **2011**, *4*, 106. (e) Singh, H. P.; Pandeya, S. N.; Chauhan, C. S.; Sharma, C. S. *Med. Chem. Res.* **2011**, *20*, 74. (f) Cotelle, N.; Hapiot, P.; Pinson, J.; Rolando, C.; Vézin, H. *J. Phys. Chem. B* **2005**, *109*, 23720. (g) Costantino, L.; Rastelli, G.; Gamberini, M. C.; Vinson, J. A.; Bose, P.; Iannone, A.; Staffieri, M.; Antolini, L.; Del Corso, A.; Mura, U.; Albasini, A. *J. Med. Chem.* **1999**, *42*, 1881.

Results

Synthesis of compound 1-13

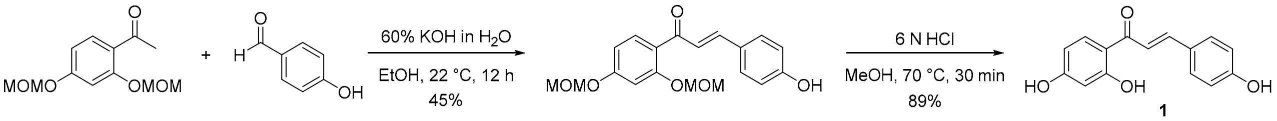

(*E*)-1-(2,4-Dihydroxyphenyl)-3-(4-hydroxyphenyl)prop-2-en-1-one (Isoliquiritigenin,1). **^{1}H NMR** (DMSO-*d$_6$*, 400 MHz): δ 13.61 (s, 1H), 10.85 (s, 1H), 10.14 (s, 1H), 8.16 (d, *J* = 8.7 Hz, 1H), 7.79-7.71 (m, 4H), 6.85 (d, *J* = 8.2 Hz, 2H), 6.40 (dd, *J* = 8.9, 2.1 Hz, 1H), 6.27 (d, *J* = 1.8 Hz, 1H); **^{13}C NMR** (DMSO-*d$_6$*, 100 MHz): δ 191.3, 165.5, 164.7, 160.0, 143.9, 132.5, 130.8, 125.6, 117.4, 115.7, 112.9, 107.9, 102.4 (Figure S1).

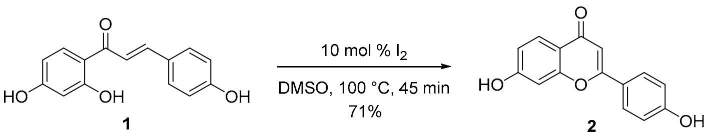

7-Hydroxy-2-(4-hydroxyphenyl)-4H-chromen-4-one (2). **^{1}H NMR** (DMSO-*d$_6$*, 400 MHz): δ 10.78 (s, 1H), 10.27 (s, 1H), 7.91 (d, *J* = 9.1 Hz, 2H), 7.85 (d, *J* = 8.7 Hz, 1H), 6.96-6.91 (m, 4H), 6.72 (s, 1H); **^{13}C NMR** (DMSO-*d$_6$*, 100 MHz): δ 176.0, 162.3, 160.5, 157.2, 127.9, 127.8, 126.2, 121.7, 116.0, 115.7, 114.5, 104.4, 102.3 (Figure S2).

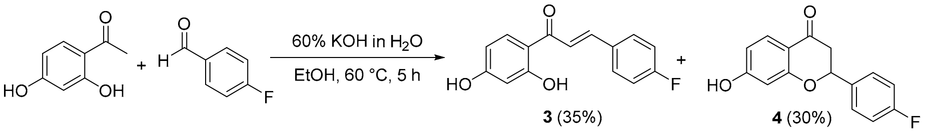

(*E*)-1-(2,4-Dihydroxyphenyl)-3-(4-fluorophenyl)prop-2-en-1-one (3). **^{1}H NMR** (DMSO-*d$_6$*, 400 MHz): δ 13.27 (s, 1H), 10.59 (s, 1H), 8.15 (d, *J* = 8.7 Hz, 1H), 7.97-7.87 (m, 3H), 7.78 (d, *J* = 15.5 Hz, 1H), 7.28 (t, *J* = 6.8 Hz, 2H), 6.43 (dd, *J* = 9.1, 2.3 Hz, 1H), 6.31 (d, *J* = 2.3 Hz, 1H); **^{13}C NMR** (DMSO-*d$_6$*, 100 MHz): δ 191.3, 165.6, 165.1, 163.2 (d, $J_{C\text{-}F}$ = 250 Hz), 142.0, 132.9, 131.1 (d, $J_{C\text{-}F}$ = 11.5 Hz), 131.1, 121.2, 115.8 (d, $J_{C\text{-}F}$ = 21.2 Hz), 112.9, 108.0, 102.5 (Figure S3).

2-(4-Fluorophenyl)-7-hydroxychroman-4-one (4). **^{1}H NMR** ($CDCl_3$, 400 MHz): δ 7.88 (d, *J* = 8.7 Hz, 1H), 7.46 (dd, *J* = 8.5, 5.3 Hz, 2H), 7.13 (t, *J* = 8.5 Hz, 2H), 6.56 (dd, *J* = 8.7, 2.3 Hz, 1H), 6.47 (d, *J* = 2.3 Hz, 1H), 5.46 (dd, *J* = 13.3, 2.7 Hz, 1H), 5.39 (s, 1H), 3.02 (dd, *J* = 16.9, 13.3 Hz, 1H), 2.83 (dd, *J* = 16.9, 3.2 Hz, 1H); **^{13}C NMR** (DMSO-*d$_6$*, 100 MHz): 189.3, 164.6, 162.8, 161.8 (d, $J_{C\text{-}F}$ = 245 Hz), 135.3, 128.6 (d, $J_{C\text{-}F}$ = 8.7 Hz), 128.2, 115.1 (d, $J_{C\text{-}F}$ = 21.2 Hz), 113.4, 110.6,

102.5, 78.1, 43.1 (Figure S4).

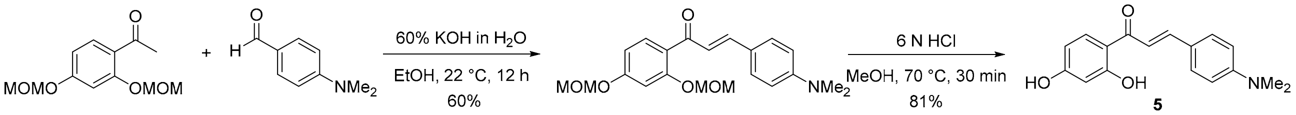

(*E*)-1-(2,4-Dihydroxyphenyl)-3-(4-(dimethylamino)phenyl)prop-2-en-1-one (5). **^{1}H NMR** (DMSO-*d*$_6$, 400 MHz): δ 13.69 (s, 1H), 10.42 (s, 1H), 8.10 (d, *J* = 8.7 Hz, 1H), 7.77-7.60 (m, 4H), 6.75 (d, *J* = 8.2 Hz, 2H), 6.40 (d, *J* = 8.7 Hz, 1H), 6.28 (s, 1H), 3.02 (s, 6H); **^{13}C NMR** (DMSO-*d*$_6$, 100 MHz): δ 191.2, 165.5, 164.4, 152.0, 144.7, 132.2, 130.7, 121.8, 114.8, 113.0, 111.6, 107.7, 102.4, 39.4 (Figure S5).

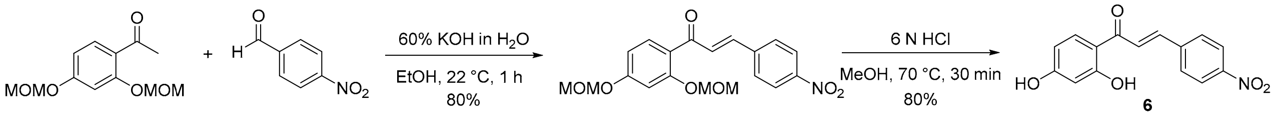

(*E*)-1-(2,4-Dihydroxyphenyl)-3-(4-nitrophenyl)prop-2-en-1-one (6). **^{1}H NMR** (DMSO-*d*$_6$, 400 MHz): δ 13.11 (s, 1H), 8.27 (d, *J* = 8.7 Hz, 2H), 8.18-8.09 (m, 4H), 7.84 (d, *J* = 15.6 Hz, 1H), 6.46 (dd, *J* = 8.9, 2.5 Hz, 1H), 6.33 (d, *J* = 2.3 Hz, 1H); **^{13}C NMR** (DMSO-*d*$_6$, 100 MHz): δ 190.7, 165.6, 165.4, 147.9, 140.9, 140.2, 133.0, 129.6, 125.6, 123.6, 113.0, 108.2, 102.5 (Figure S6).

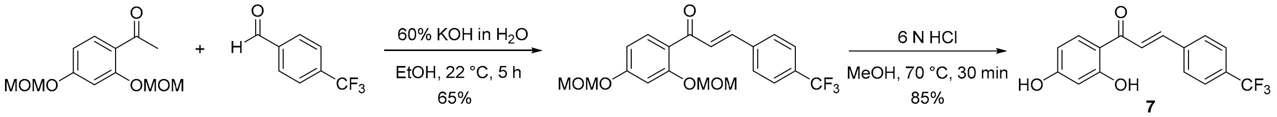

(*E*)-1-(2,4-Dihydroxyphenyl)-3-(4-(trifluoromethyl)phenyl)prop-2-en-1-one (7). **^{1}H NMR** (DMSO-*d*$_6$, 400 MHz): δ 13.12 (s, 1H), 10.64 (s, 1H), 8.17 (d, *J* = 8.7 Hz, 1H), 8.10-8.04 (m, 3H), 7.84-7.79 (m, 3H), 6.45 (dd, *J* = 8.7, 2.3 Hz, 1H), 6.32 (d, *J* = 2.3 Hz, 1H); **^{13}C NMR** (DMSO-*d*$_6$, 100 MHz): δ 191.0, 165.4, 165.3, 141.1, 138.5, 133.0, 129.9 (q, $J_{C\text{-}F}$ = 31.8 Hz), 129.2, 125.4 (q, $J_{C\text{-}F}$ = 3.9 Hz), 124.2, 123.8 (q, $J_{C\text{-}F}$ = 272 Hz), 113.0, 108.2, 102.5 (Figure S7).

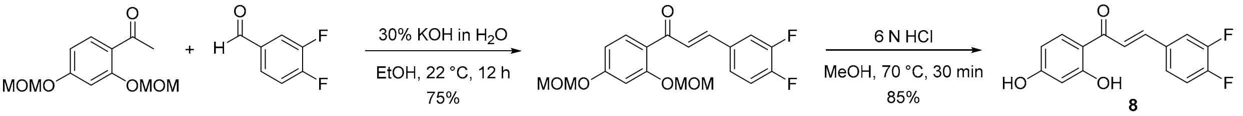

(*E*)-3-(3,4-Difluorophenyl)-1-(2,4-dihydroxyphenyl)prop-2-en-1-one (8). **^{1}H NMR** (DMSO-*d*$_6$, 400 MHz): δ 13.2 (s, 1H), 8.17 (d, *J* = 9.1 Hz, 1H), 8.07 (ddd, *J* = 12.1, 8.0, 1.8 Hz, 1H), 7.94 (d, *J* = 15.6 Hz, 1H), 7.75-7.69 (m, 2H), 7.49 (dt,

J = 10.5, 8.5 Hz, 1H), 6.43 (dd, J = 9.1, 2.3 Hz, 1H), 6.31 (d, J = 2.3 Hz, 1H); **^{13}C NMR** (DMSO-d_6, 100 MHz): δ 191.1, 165.6, 165.2, 150.5 (dd, J_{C-F} = 250 Hz, 13.4 Hz), 149.6 (dd, J_{C-F} = 246, 12.5 Hz), 140.9, 132.95, 132.4 (dd, J_{C-F} = 5.8, 3.9 Hz), 126.5 (dd, J_{C-F} = 6.7, 3.9 Hz), 122.7, 117.7 (d_{C-F}, J = 18.3 Hz), 116.8 (d, J_{C-F} = 17.3 Hz), 112.9, 108.1, 102.4 (Figure S8).

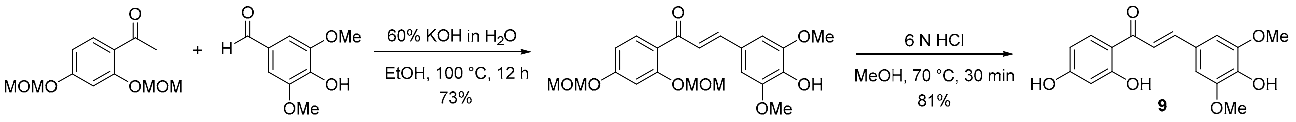

(*E*)-1-(2,4-Dihydroxyphenyl)-3-(4-hydroxy-3,5-dimethoxyphenyl)prop-2-en-1-one (9). **^{1}H NMR** (DMSO-d_6, 400 MHz): δ 13.53 (s, 1H), 10.52 (s, 1H), 8.88 (s, 1H), 8.18 (d, J = 8.7 Hz, 1H), 7.78 (d, J = 15.1 Hz, 1H), 7.73 (d, J = 15.5 Hz, 1H), 7.19 (s, 2H), 6.43 (dd, J = 8.7, 2.3 Hz, 1H), 6.30 (d, J = 2.3 Hz, 1H), 3.86 (s, 6H); **^{13}C NMR** (DMSO-d_6, 100 MHz): δ 191.4, 165.6, 164.7, 148.1, 144.7, 139.0, 132.6, 124.9, 118.0, 112.9, 107.8, 107.3, 102.5, 56.2 (Figure S9).

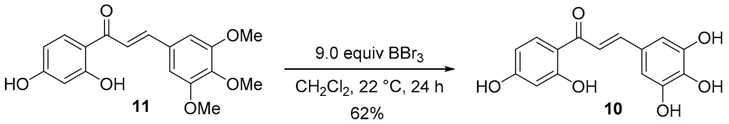

(*E*)-1-(2,4-Dihydroxyphenyl)-3-(3,4,5-trihydroxyphenyl)prop-2-en-1-one (10). **^{1}H NMR** (DMSO-d_6, 400 MHz): δ 13.43 (s, 1H), 10.53 (s, 1H), 8.95 (s, 2H), 8.78 (s, 1H), 8.05 (d, J = 9.1 Hz, 1H), 7.57 (d, J = 15.6, 1H), 7.52 (d, J = 15.6, 1H), 6.80 (s, 2H), 6.41 (dd, J = 9.1, 2.3 Hz, 1H), 6.29 (d, J = 2.3 Hz, 1H); **^{13}C NMR** (DMSO-d_6, 100 MHz): δ 191.2, 165.4, 164.7, 146.0, 144.8, 136.9, 132.4, 125.1, 117.4, 113.0, 108.5, 108.0, 102.5 (Figure S10).

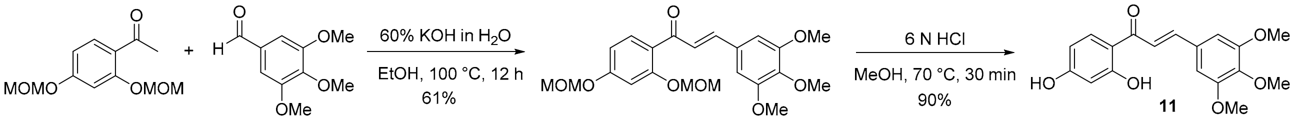

(*E*)-1-(2,4-Dihydroxyphenyl)-3-(3,4,5-trimethoxyphenyl)prop-2-en-1-one (11). **^{1}H NMR** (DMSO-d_6, 400 MHz): δ 13.38 (s, 1H), 10.60 (s, 1H), 8.19 (d, J = 9.1 Hz, 1H), 7.87 (d, J = 15.6 Hz, 1H), 7.74 (d, J = 15.1 Hz, 1H), 7.21 (s, 2H), 6.45 (dd, J = 8.7, 2.3 Hz, 1H), 6.31 (d, J = 2.3 Hz, 1H), 3.88 (s, 6H), 3.74 (s, 3H); **^{13}C NMR** (DMSO-d_6, 100 MHz): δ 191.3, 165.6, 165.0, 153.0, 143.8, 140.1, 132.8, 129.9, 120.3, 112.9, 108.0, 106.8, 102.5, 59.9, 56.1 (Figure S11).

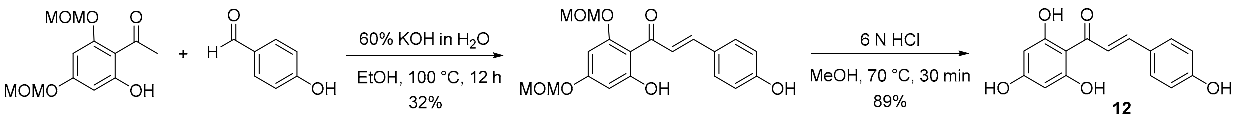

(*E*)-3-(4-Hydroxyphenyl)-1-(2,4,6-trihydroxyphenyl)prop-2-en-1-one (12). **^{1}H NMR** (DMSO-d_6, 400 MHz): δ 12.38 (s, 2H), 10.22 (s, 1H), 9.89 (s, 1H), 7.95 (d, *J* = 16.0 Hz, 1H), 7.64 (d, *J* = 15.6 Hz, 1H), 7.51 (d, *J* = 8.2 Hz, 2H), 6.84 (d, *J* = 8.7 Hz, 2H), 5.85 (s, 2H); **^{13}C NMR** (DMSO-d_6, 100 MHz): δ 191.6, 164.5, 164.1, 159.6, 141.9, 130.0, 126.0, 123.9, 115.8, 104.2, 94.8 (Figure S12).

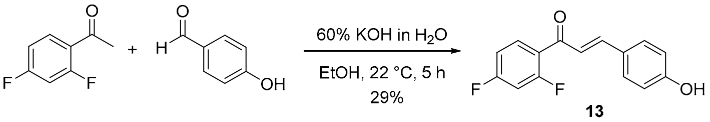

(*E*)-1-(2,4-Difluorophenyl)-3-(4-hydroxyphenyl)prop-2-en-1-one (13). **^{1}H NMR** ($CDCl_3$, 400 MHz): δ 7.89 (td, *J* = 8.6, 6.6 Hz, 1H), 7.74 (dd, *J* = 16.0, 1.8 Hz, 1H), 7.56 (d, *J* = 8.7 Hz, 2H), 7.28 (dd, *J* = 15.5, 2.7 Hz, 1H), 7.00 (td, *J* = 8.2, 2.3 Hz, 1H), 6.94-6.91 (m, 1H), 6.88 (d, *J* = 8.7 Hz, 2H), 5.13 (s, 1H); **^{13}C NMR** ($CDCl_3$, 100 MHz): δ 188.4, 165.4 (dd, $J_{C\text{-}F}$ = 256, 11.5 Hz), 161.9 (dd, $J_{C\text{-}F}$ = 256, 12.6 Hz), 158.9, 146.0, 132.8 (dd, $J_{C\text{-}F}$ = 10.6, 3.9 Hz), 130.9, 126.9, 123.5 (dd, $J_{C\text{-}F}$ = 12.5, 2.9 Hz), 122.4, 116.1, 112.22 (dd, $J_{C\text{-}F}$ = 21.2, 3.8 Hz), 104.8 (dd, $J_{C\text{-}F}$= 27.0, 26.0 Hz) (Figure S13).

Supplementary Figures 1-12. Copies of ^{1}H and ^{13}C NMR spectra for all products

Figure S1a. ^{1}H NMR spectrum of the isoliquiritigenin (1)

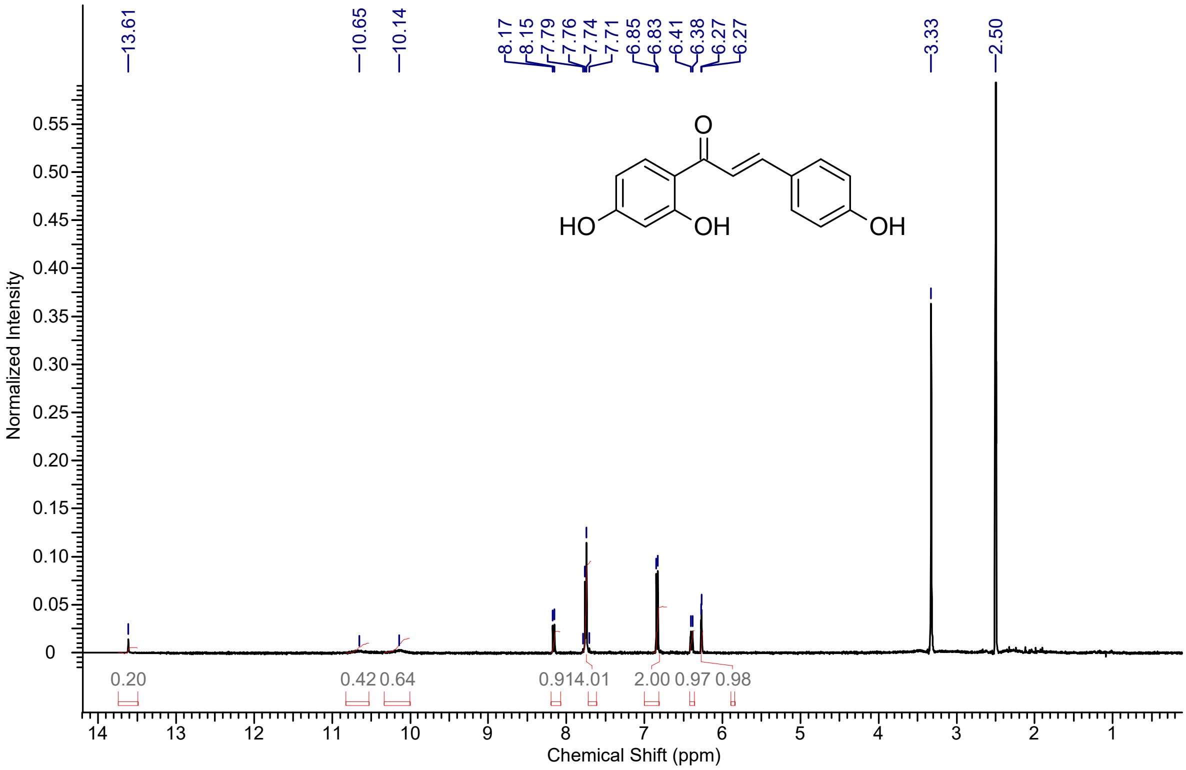

Figure S1b. ^{13}C NMR spectrum of the isoliquiritigenin **(1)**

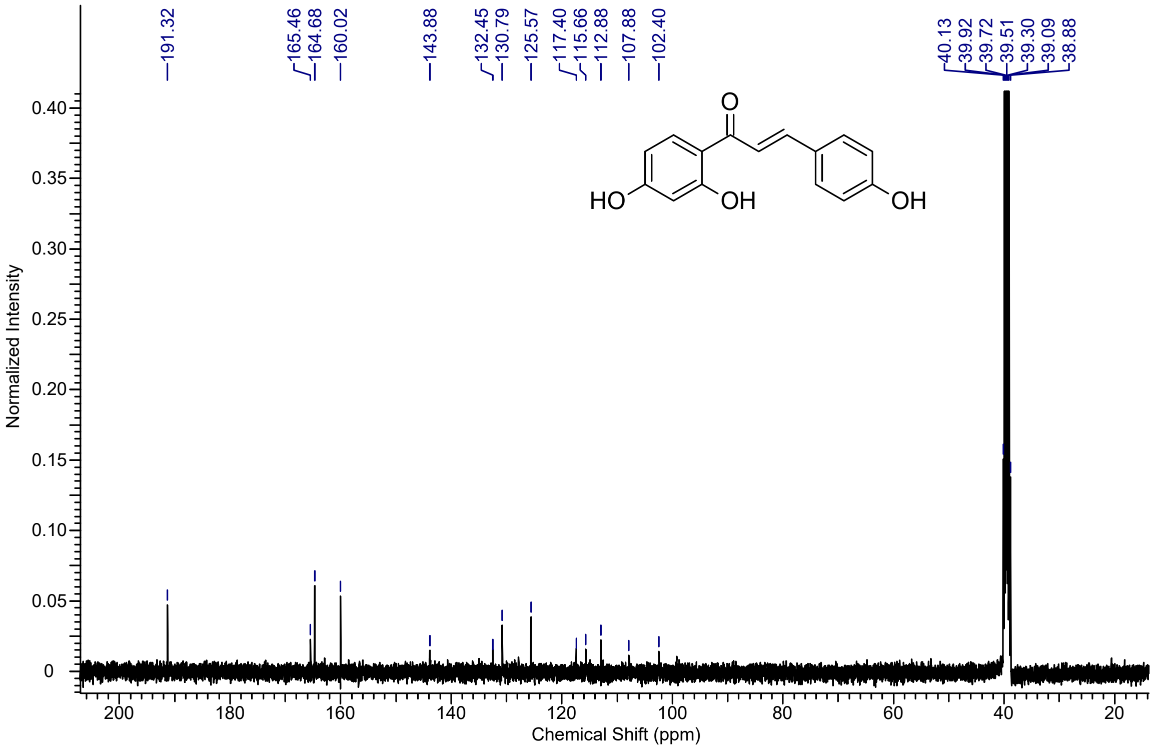

Figure S2a. ^{1}H NMR spectrum of the compound **2**

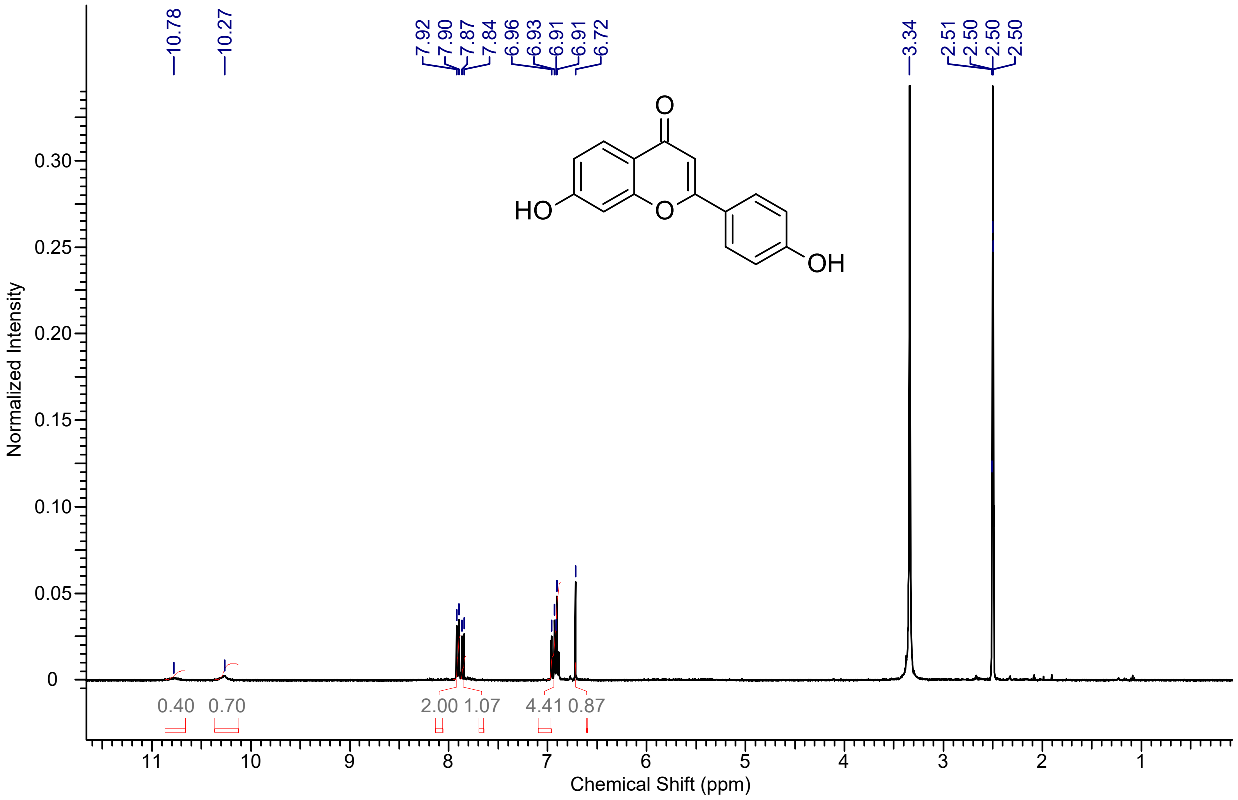

Figure S2b. ^{13}C NMR spectrum of the compound **2**

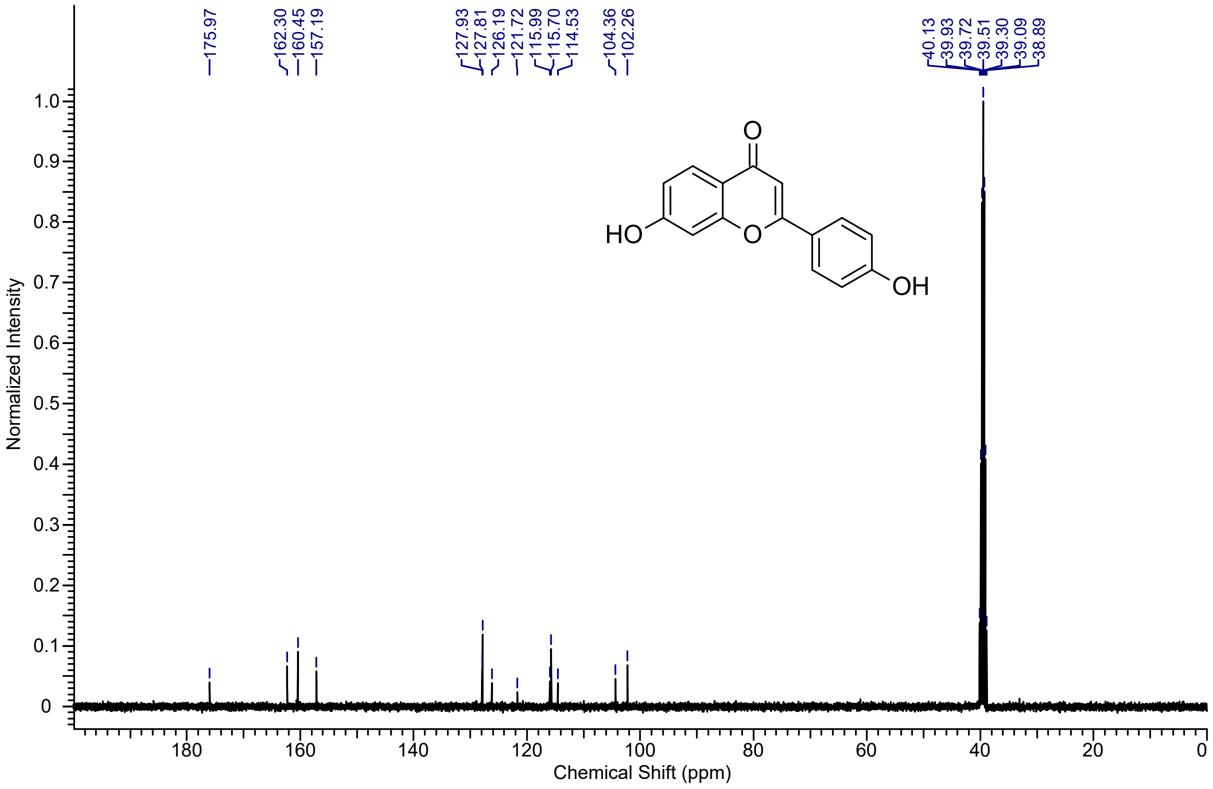

Figure S3a. ^{1}H NMR spectrum of the compound **3**

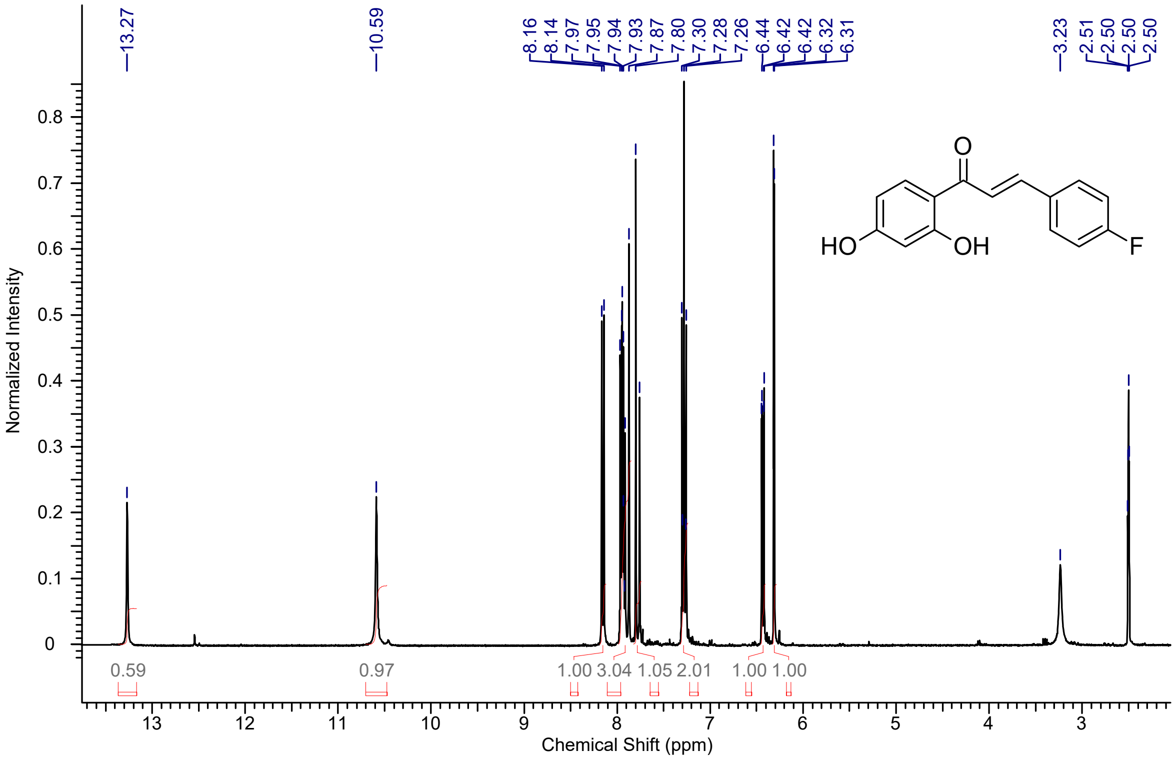

Figure S3b. ^{13}C NMR spectrum of the compound **3**

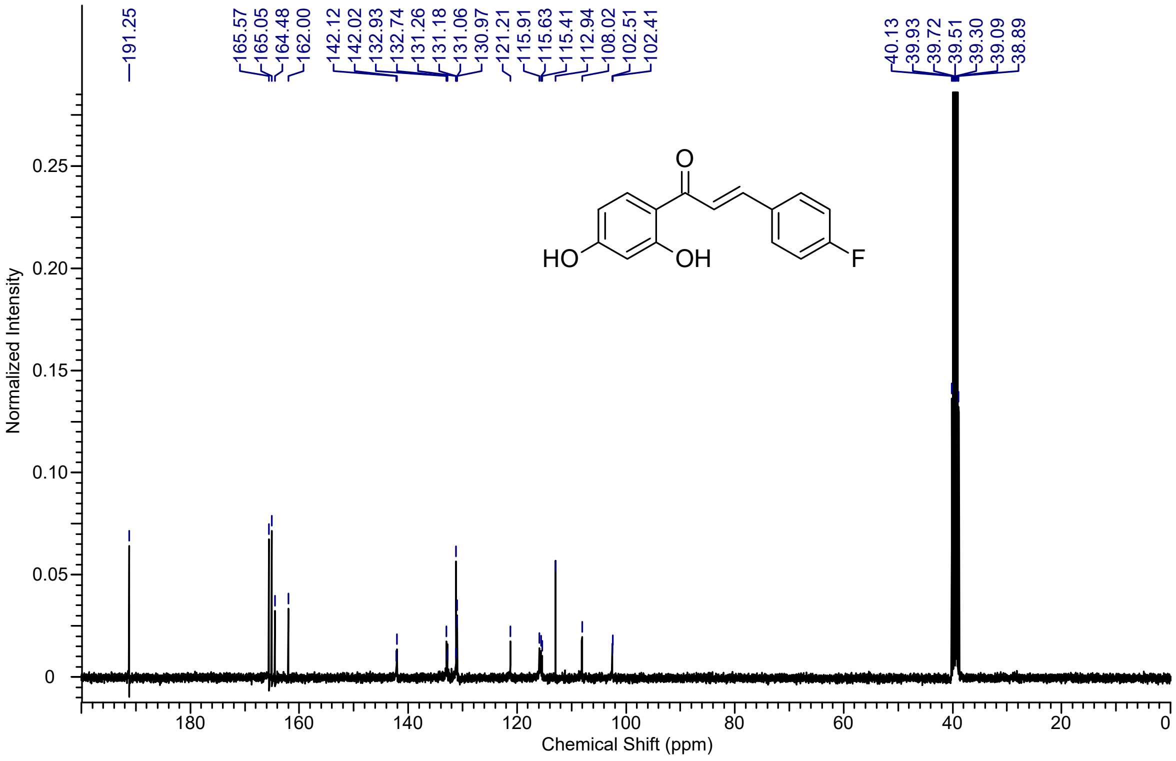

Figure S4a. ^{1}H NMR spectrum of the compound **4**

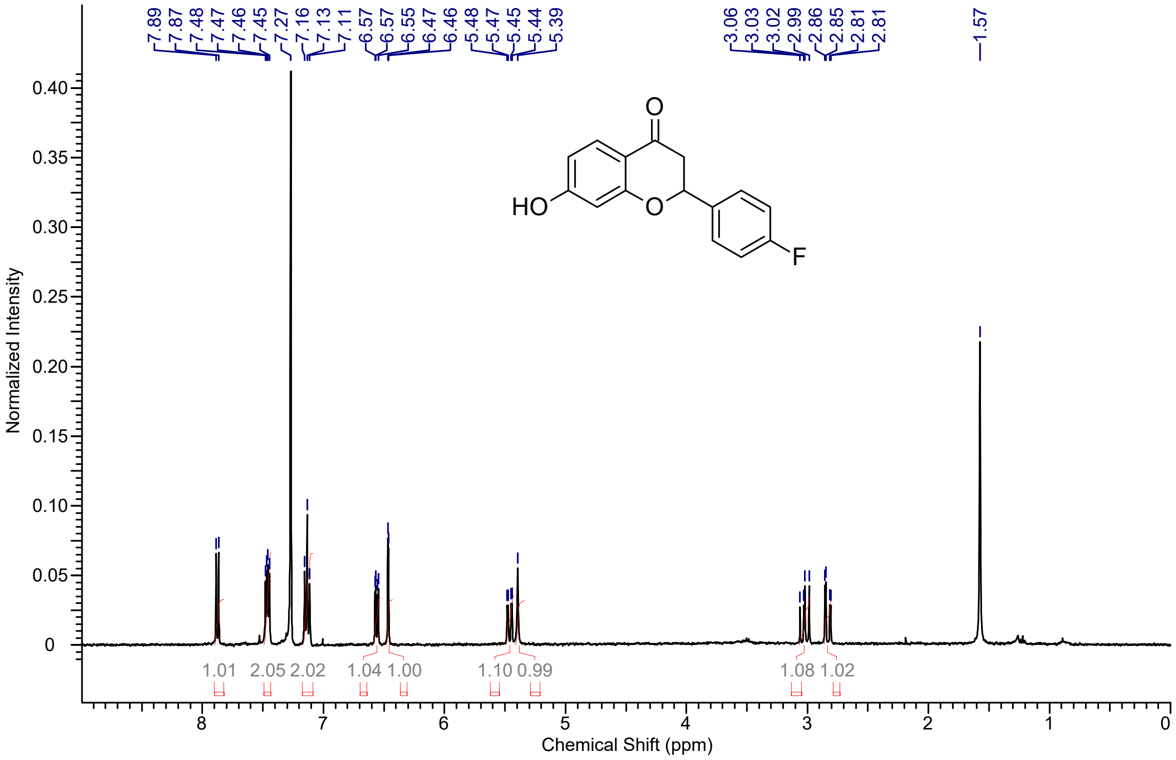

Figure S4b. ^{13}C NMR spectrum of the compound **4**

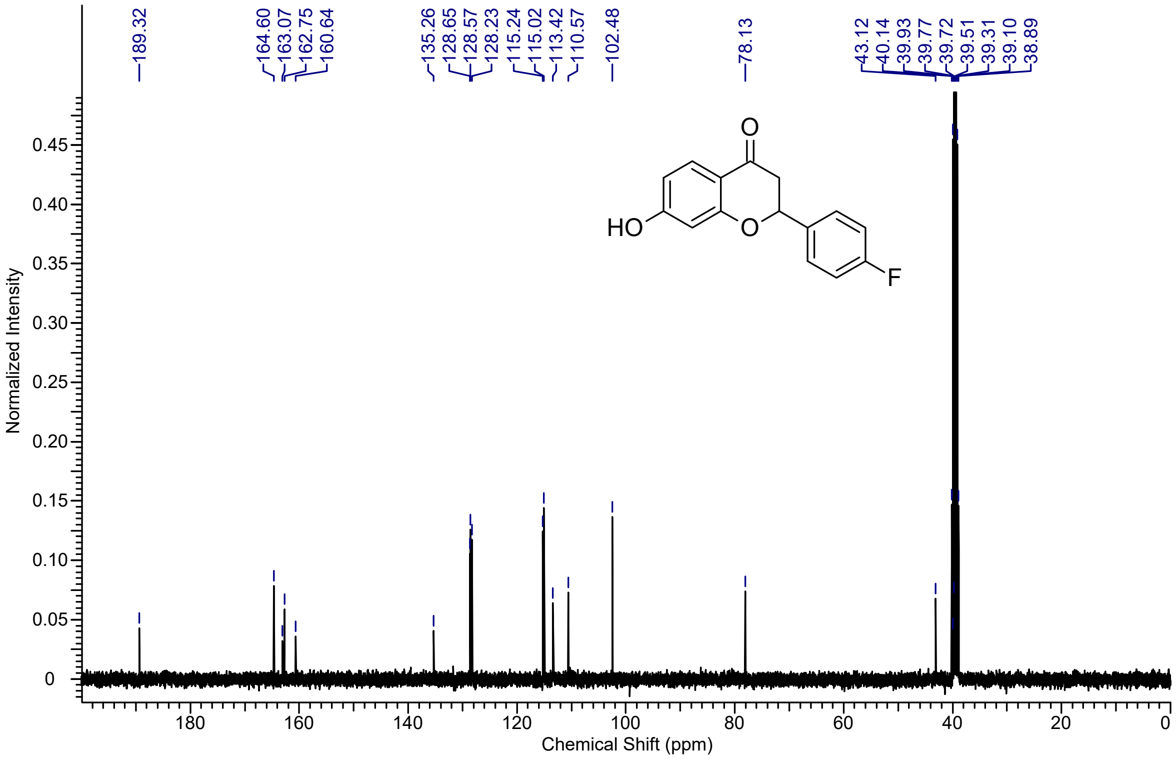

Figure S5a. ^{1}H NMR spectrum of the compound **5**

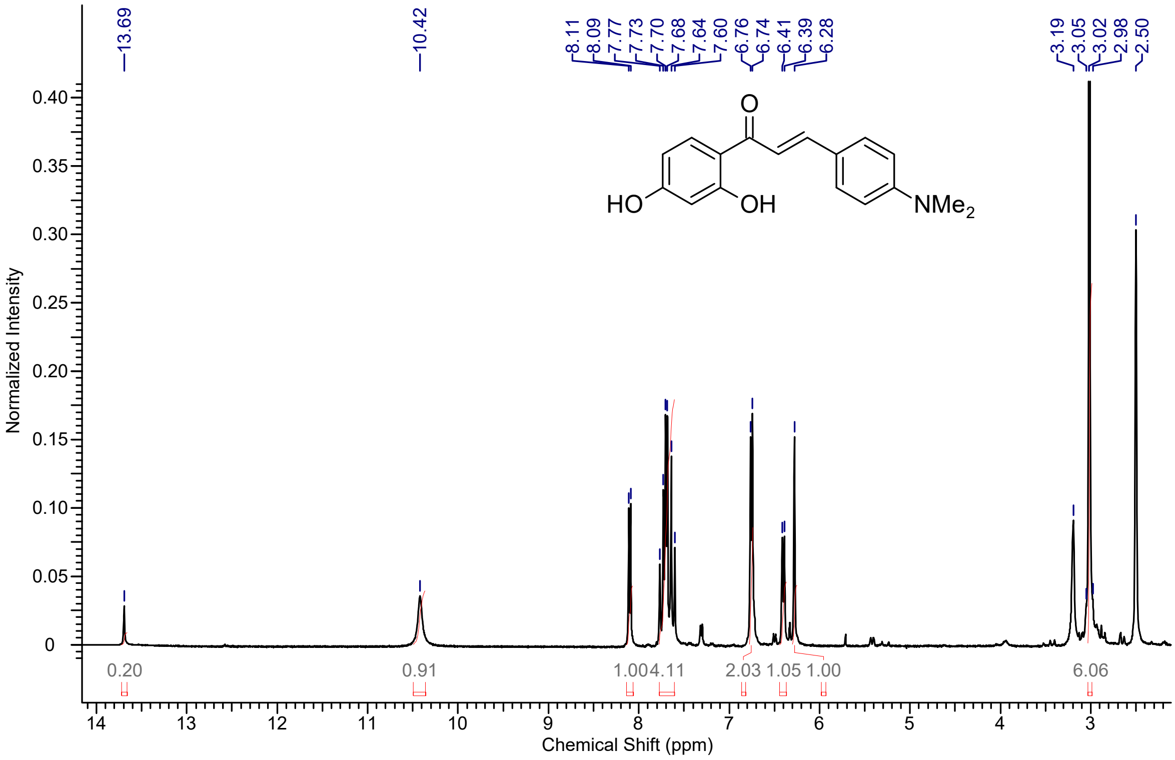

Figure S5b. ^{13}C NMR spectrum of the compound **5**

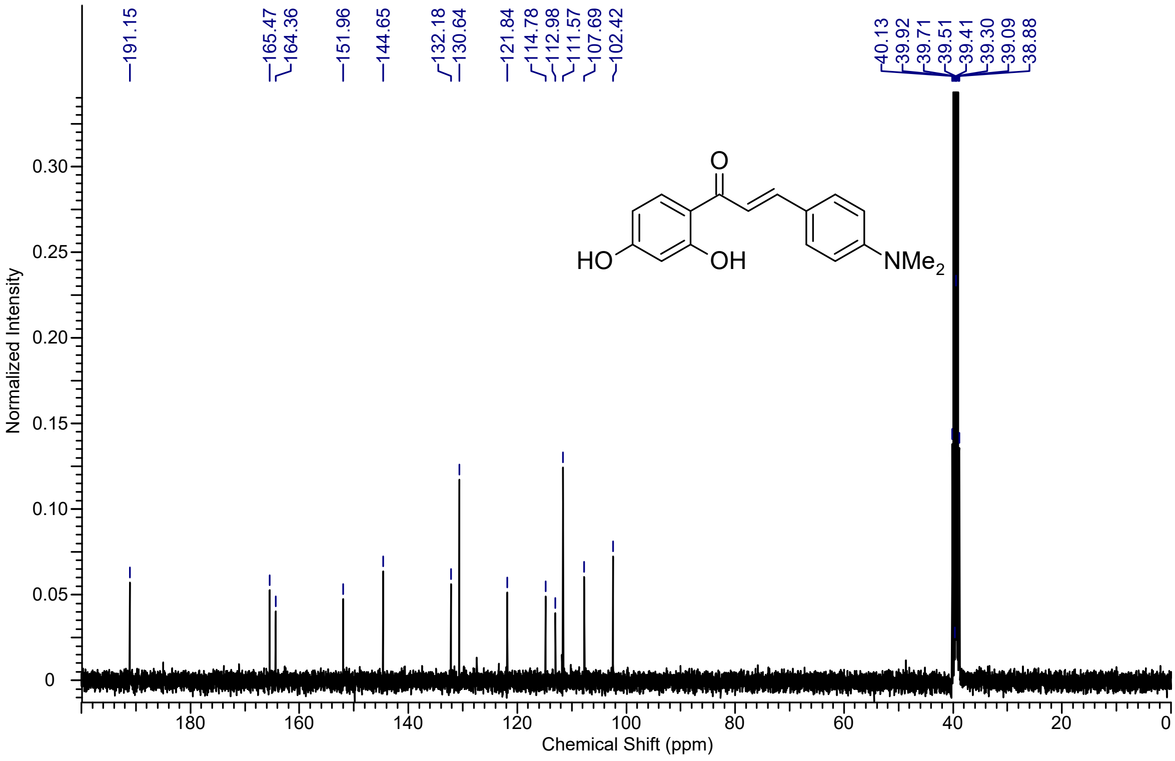

Figure S6a. ^{1}H NMR spectrum of the compound **6**

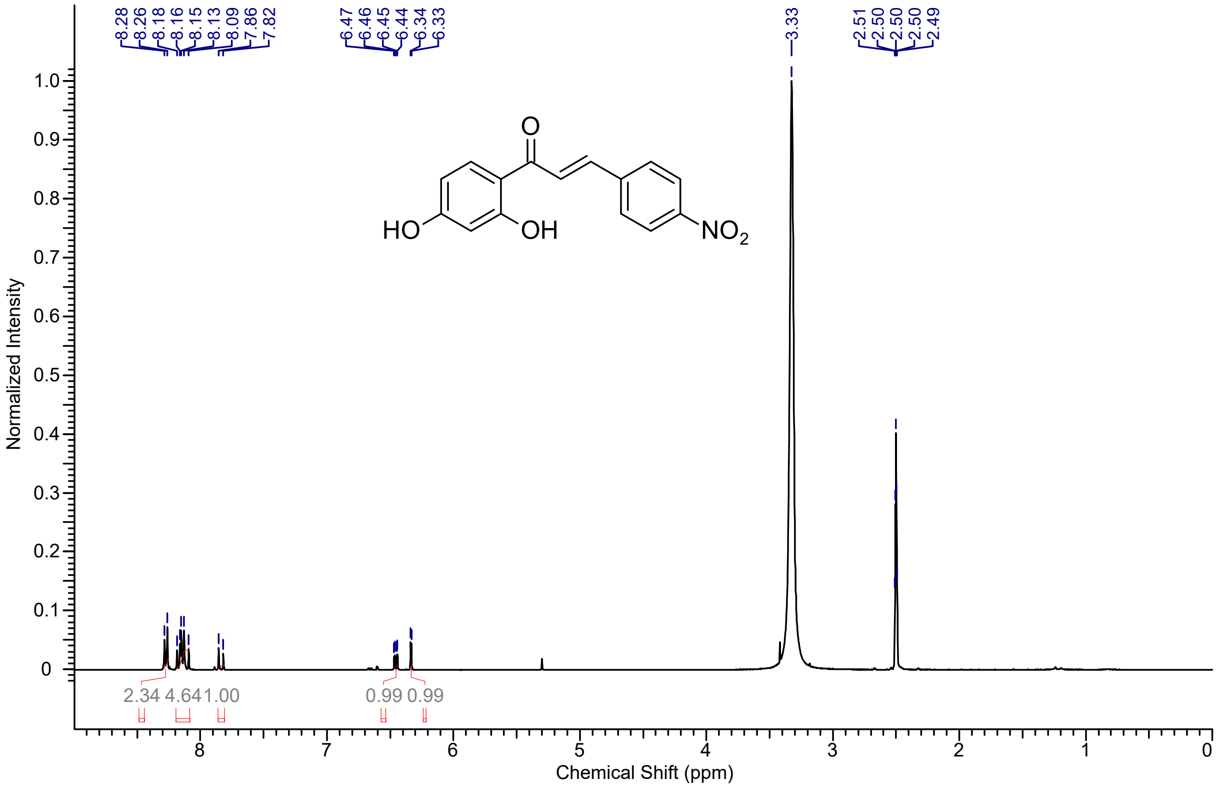

Figure S6b. ^{13}C NMR spectrum of the compound **6**

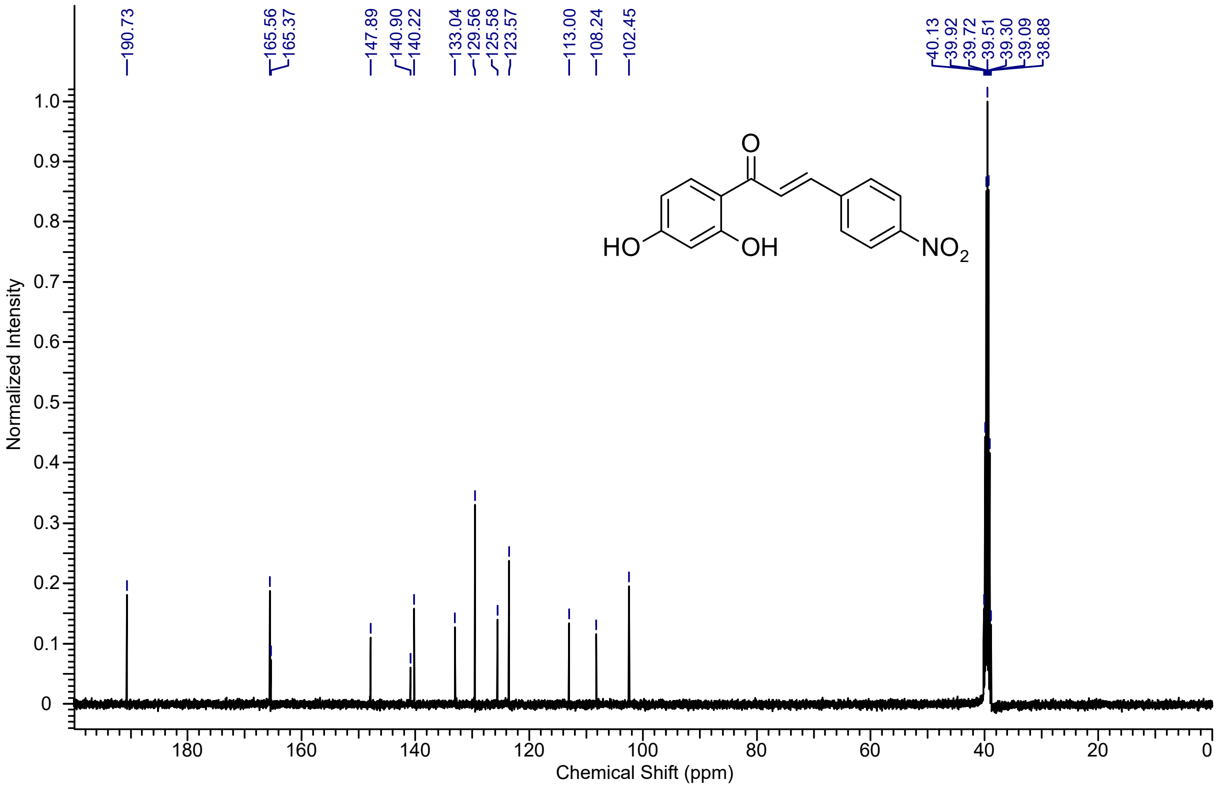

Figure S7a. ^{1}H NMR spectrum of the compound **7**

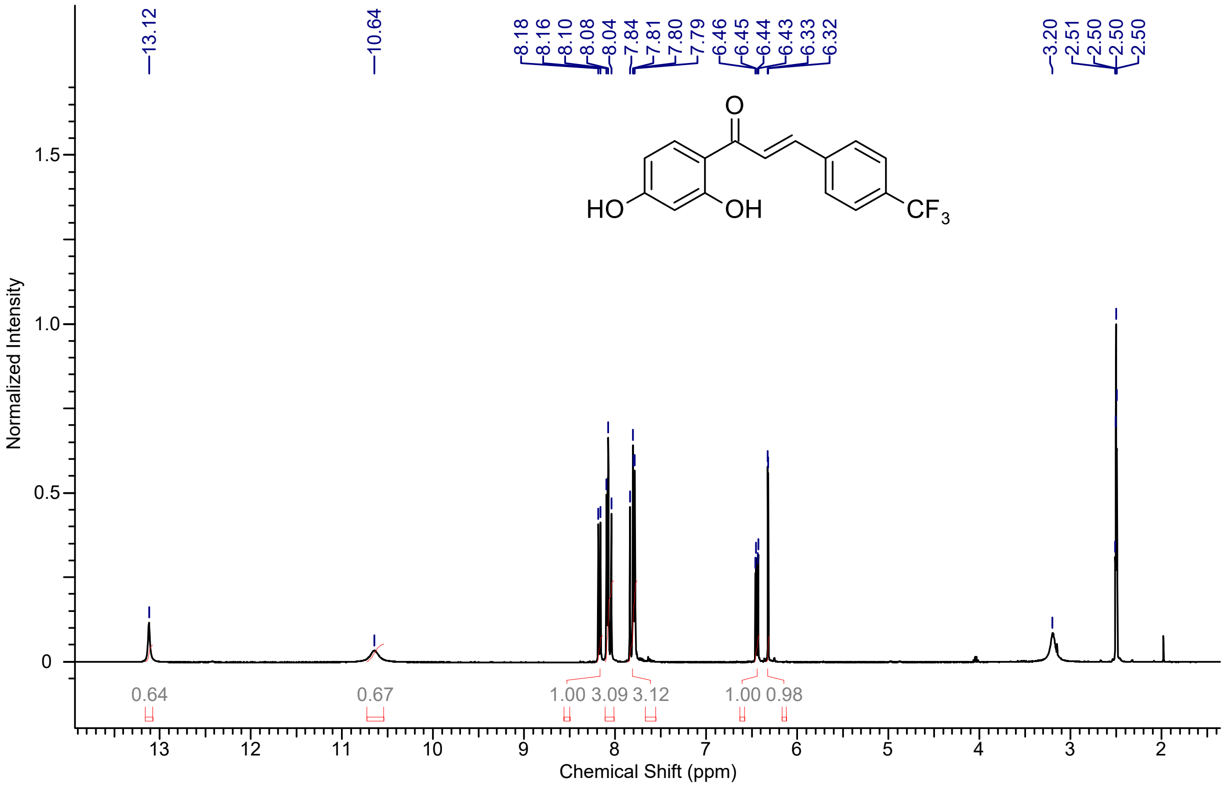

Figure S7b. ^{13}C NMR spectrum of the compound **7**

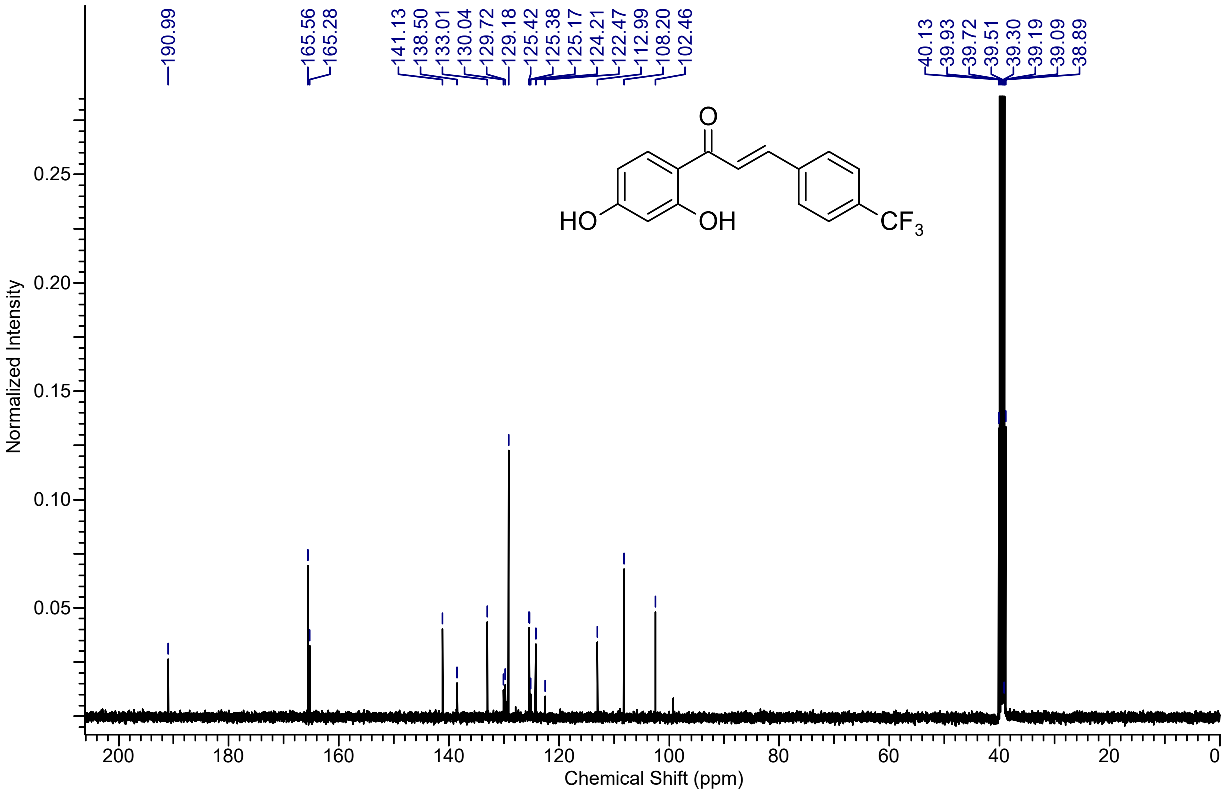

Figure S8a. ^{1}H NMR spectrum of the compound **8**

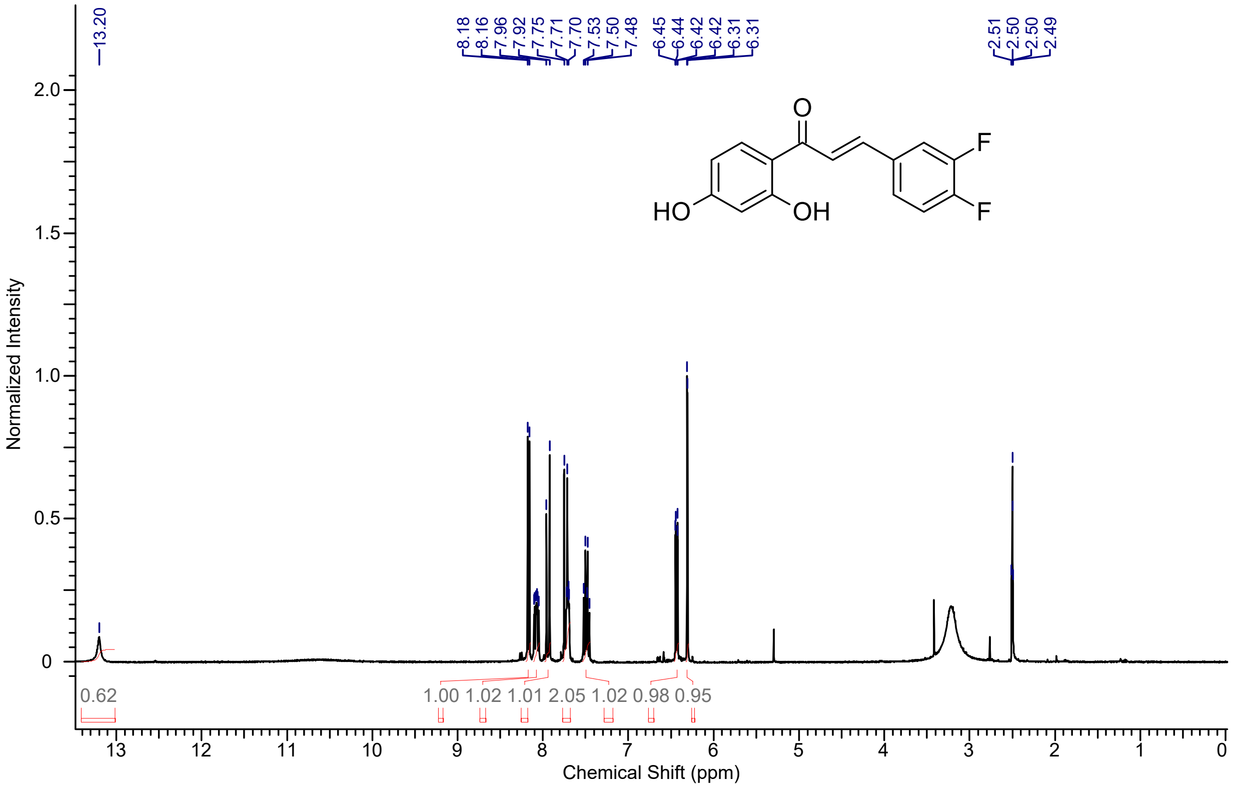

Figure S8b. ^{13}C NMR spectrum of the compound **8**

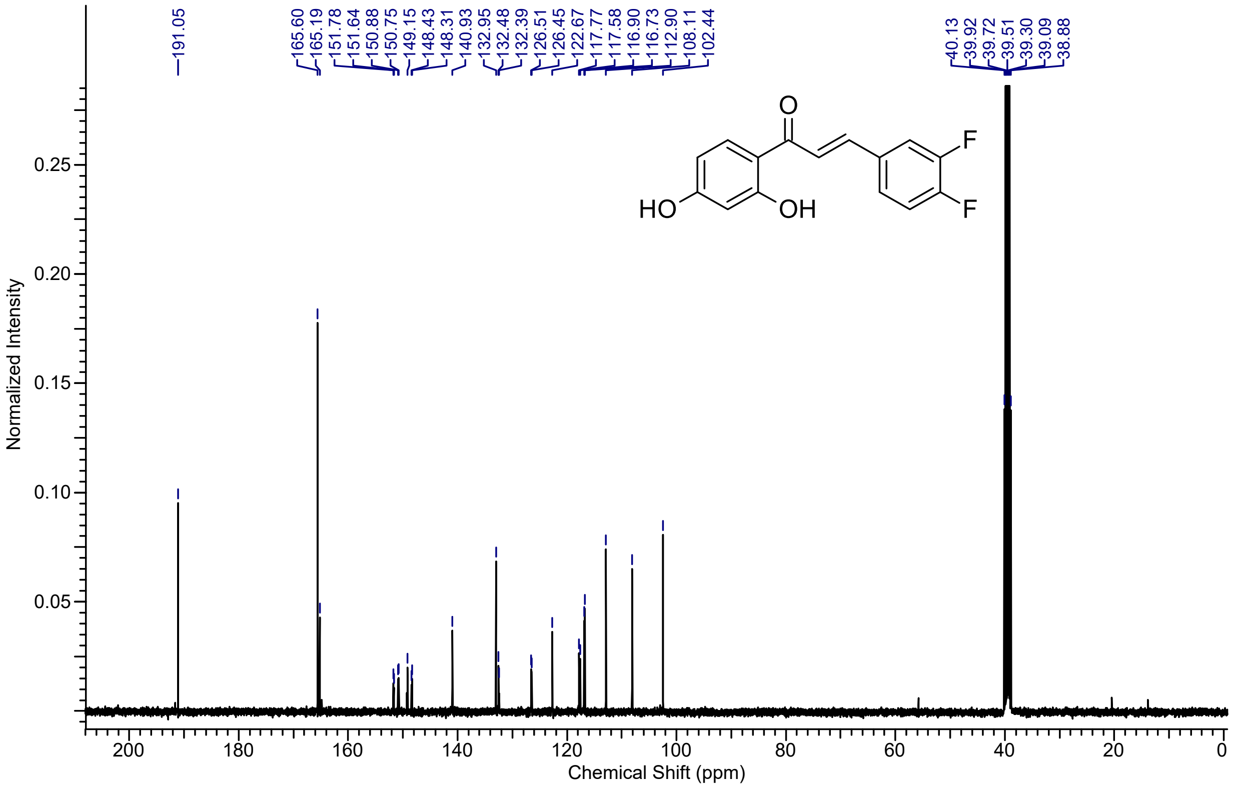

Figure S9a. ^{1}H NMR spectrum of the compound **9**

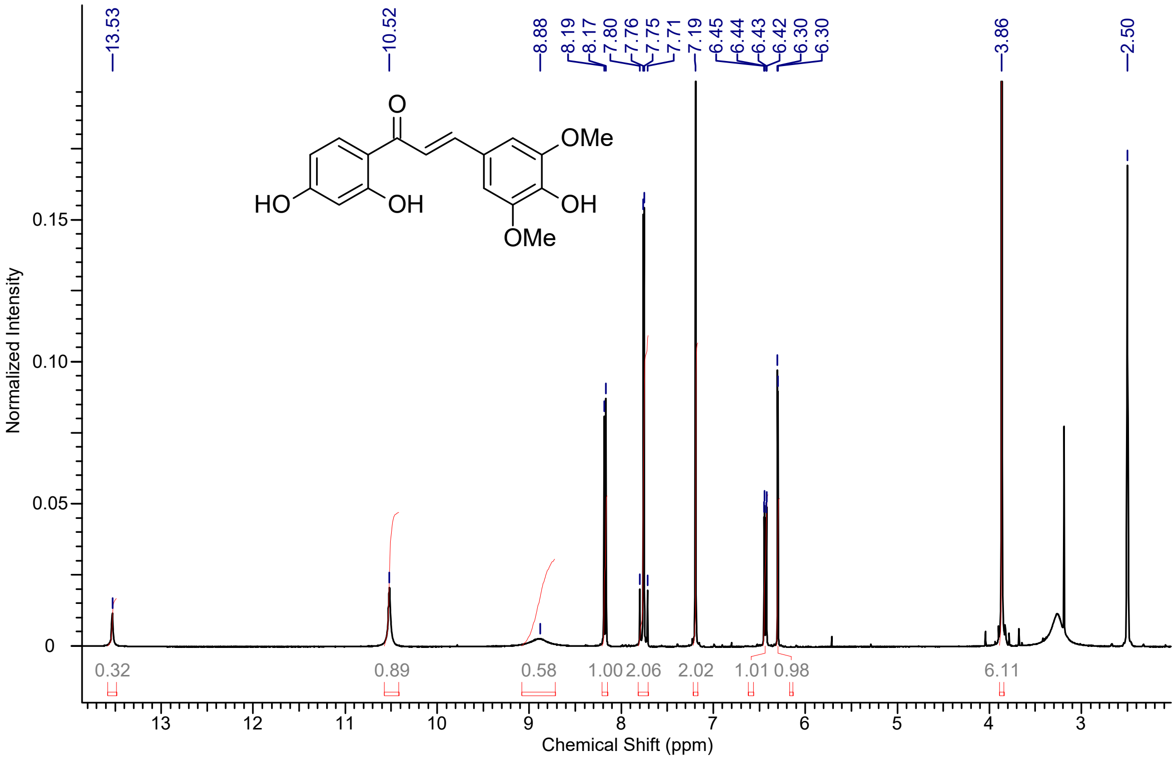

Figure S9b. ^{13}C NMR spectrum of the compound **9**

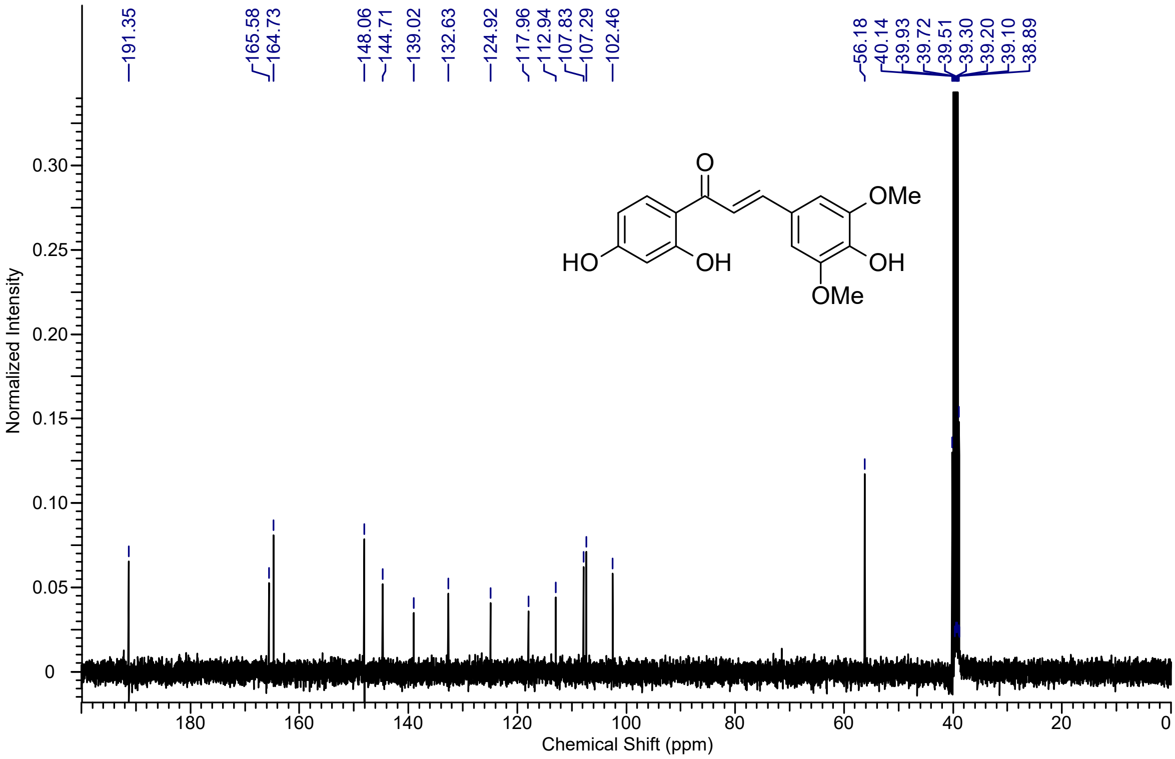

Figure S10a. ^{1}H NMR spectrum of the compound **10**

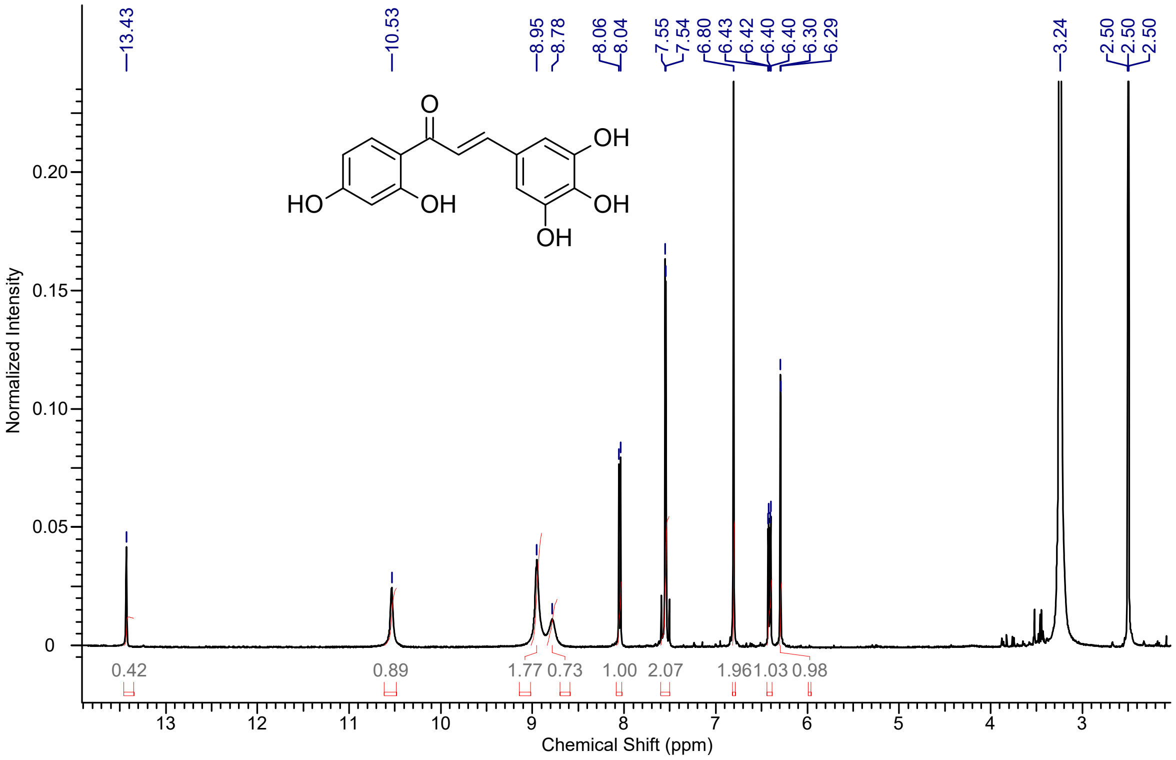

Figure S10b. ^{13}C NMR spectrum of the compound **10**

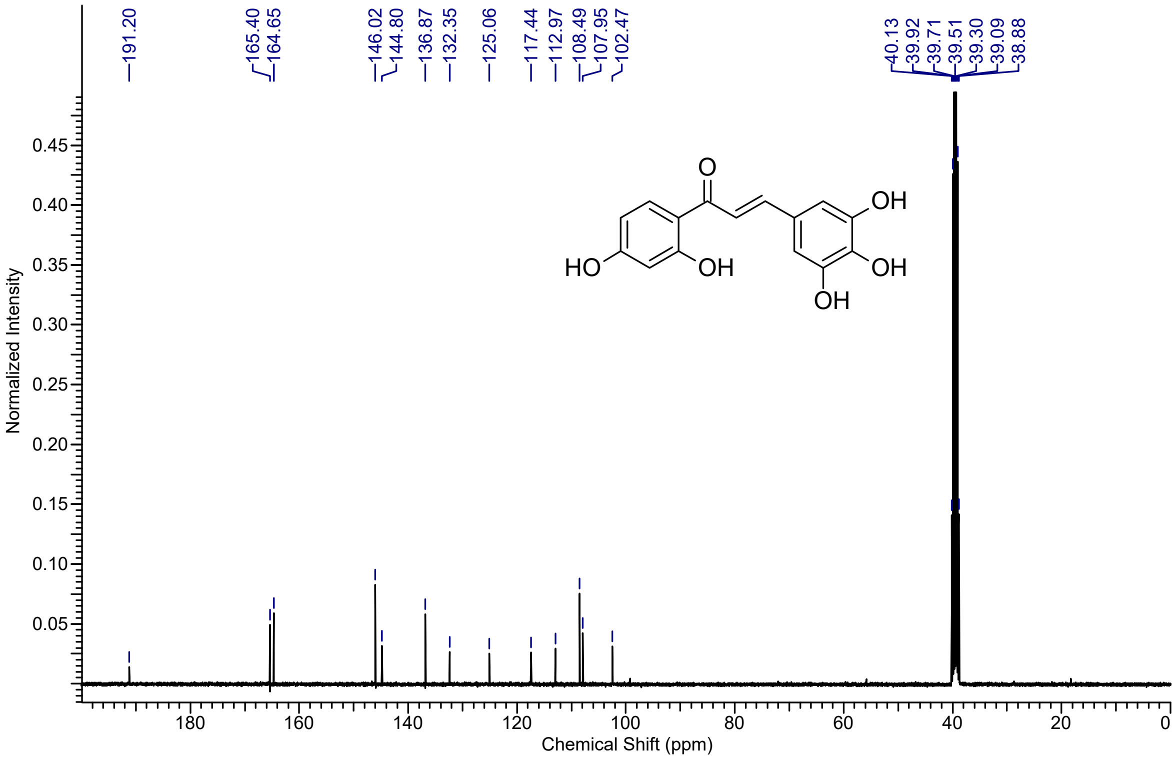

Figure S11a. ^{1}H NMR spectrum of the compound **11**

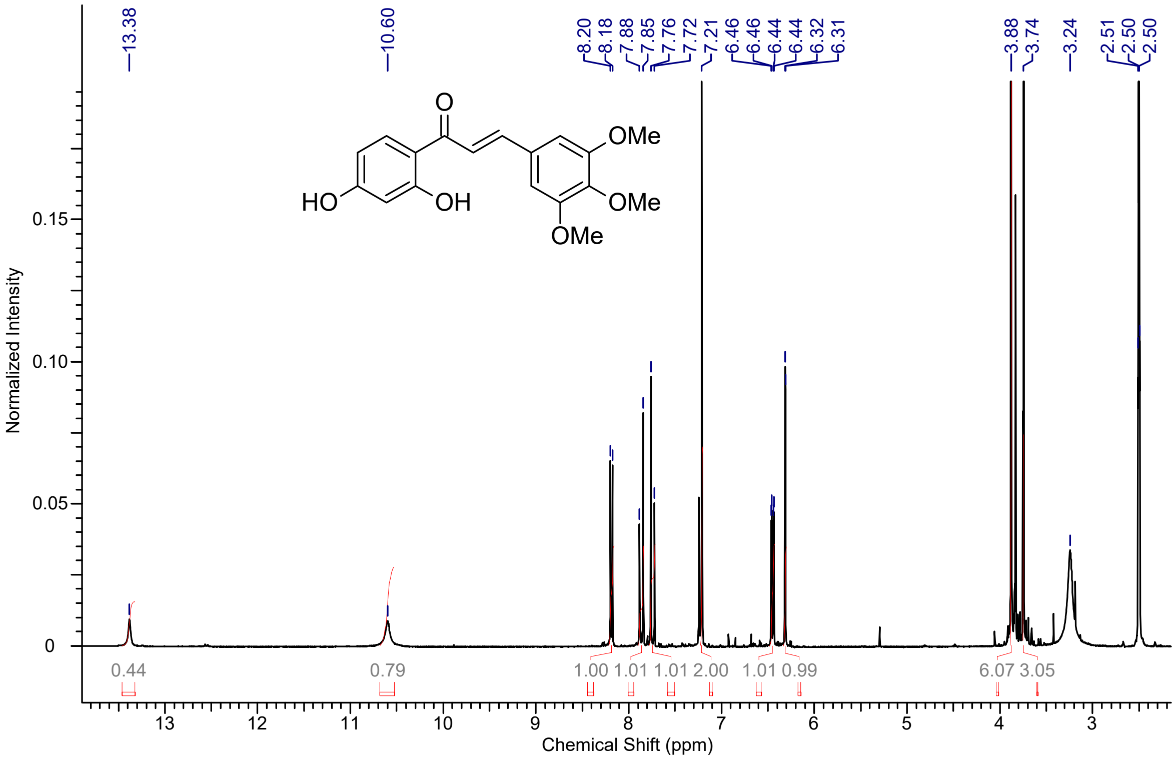

Figure S11b. ^{13}C NMR spectrum of the compound **11**

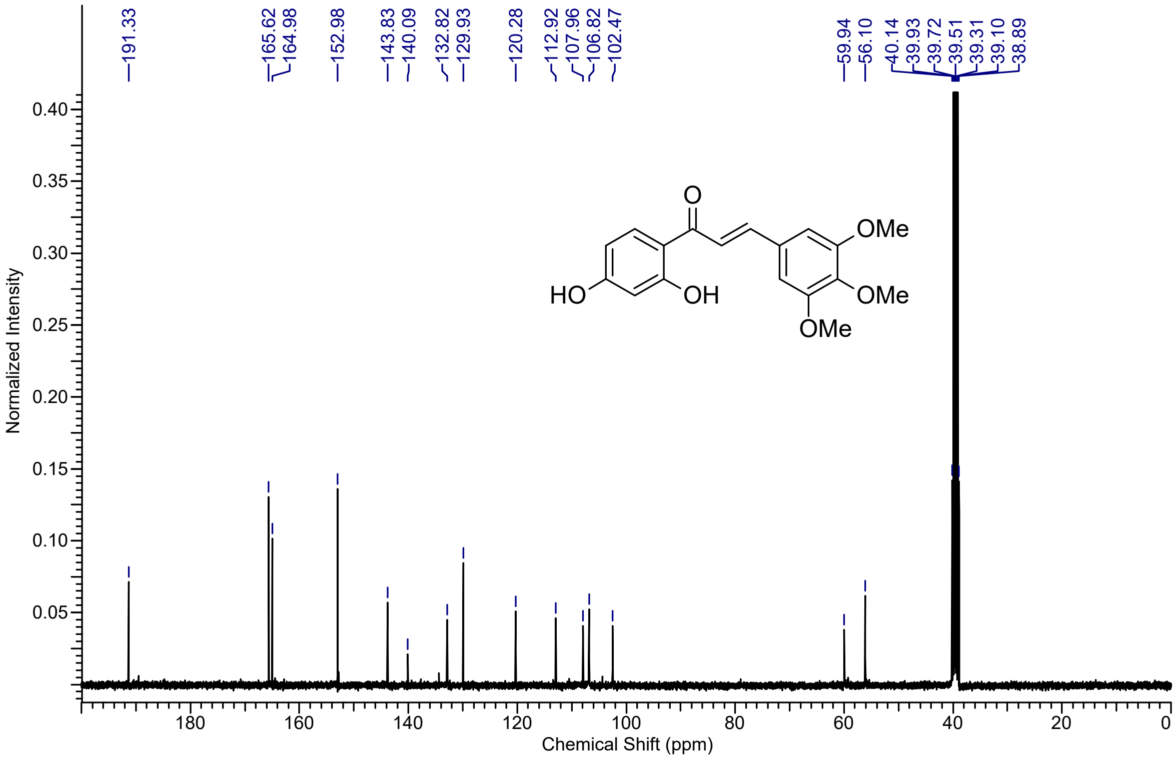

Figure S12a. ^{1}H NMR spectrum of the compound **12**

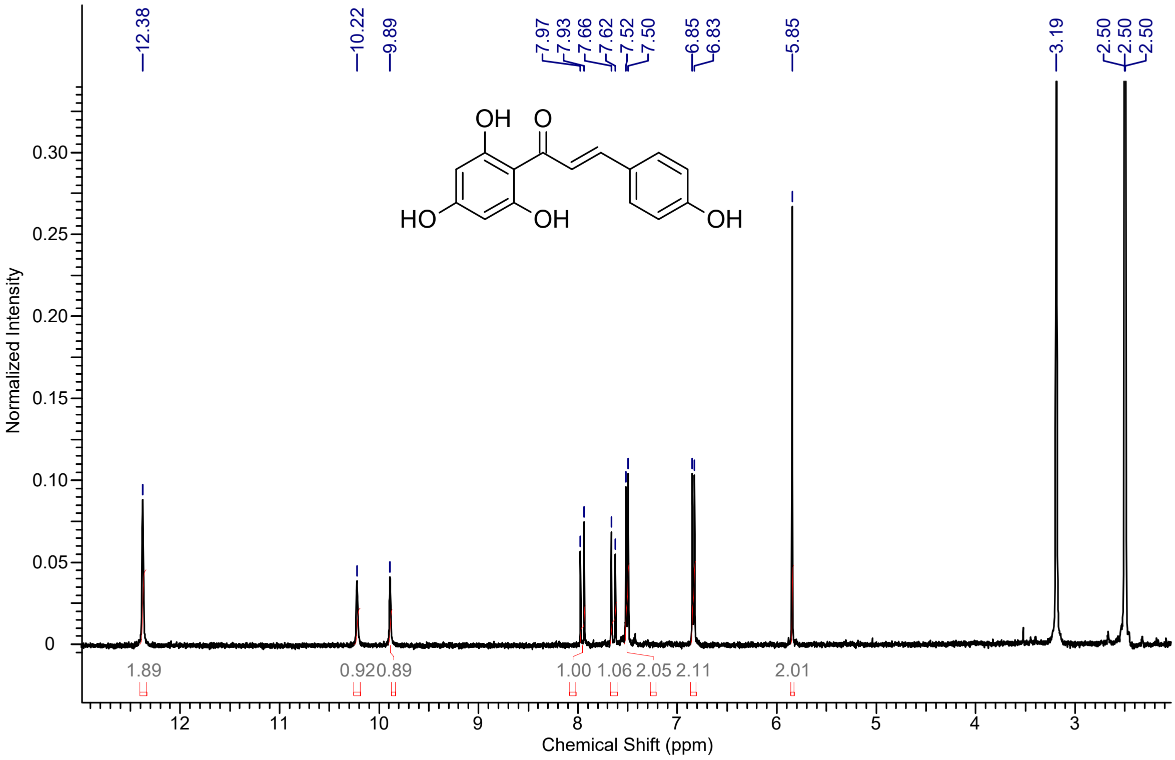

Figure S12b. ^{13}C NMR spectrum of the compound **12**

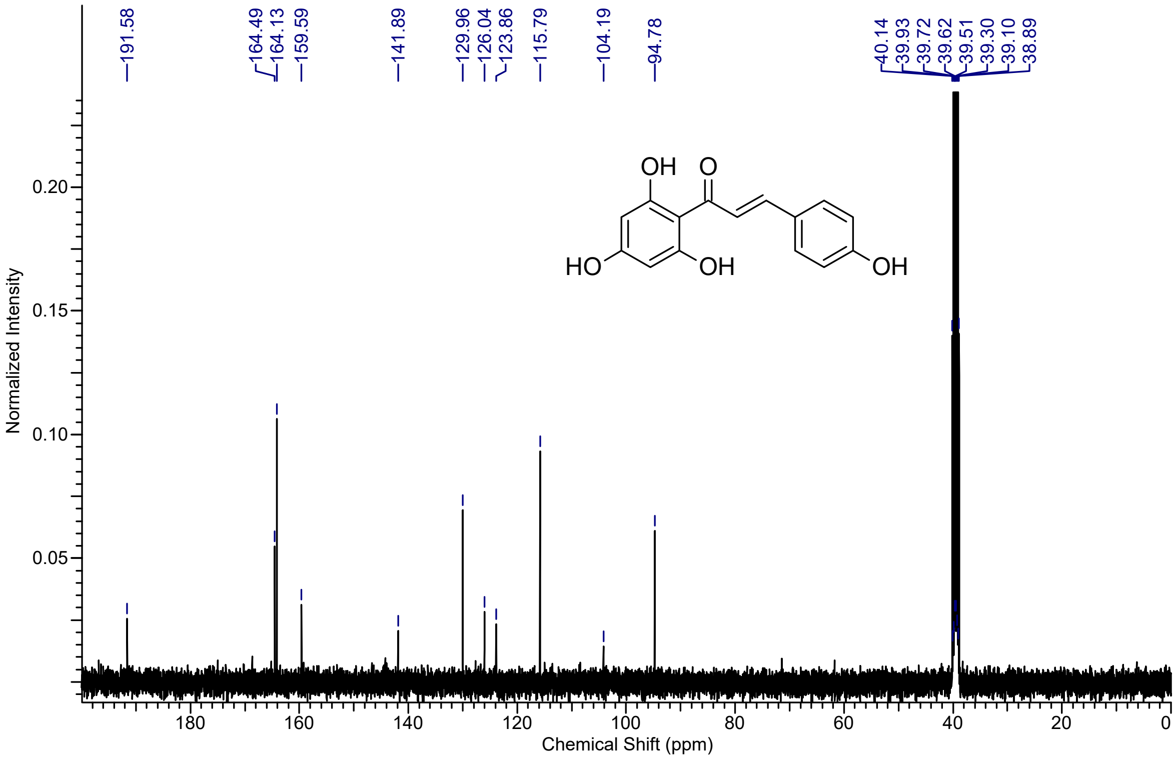

Figure S13a. ^{1}H NMR spectrum of the compound **13**

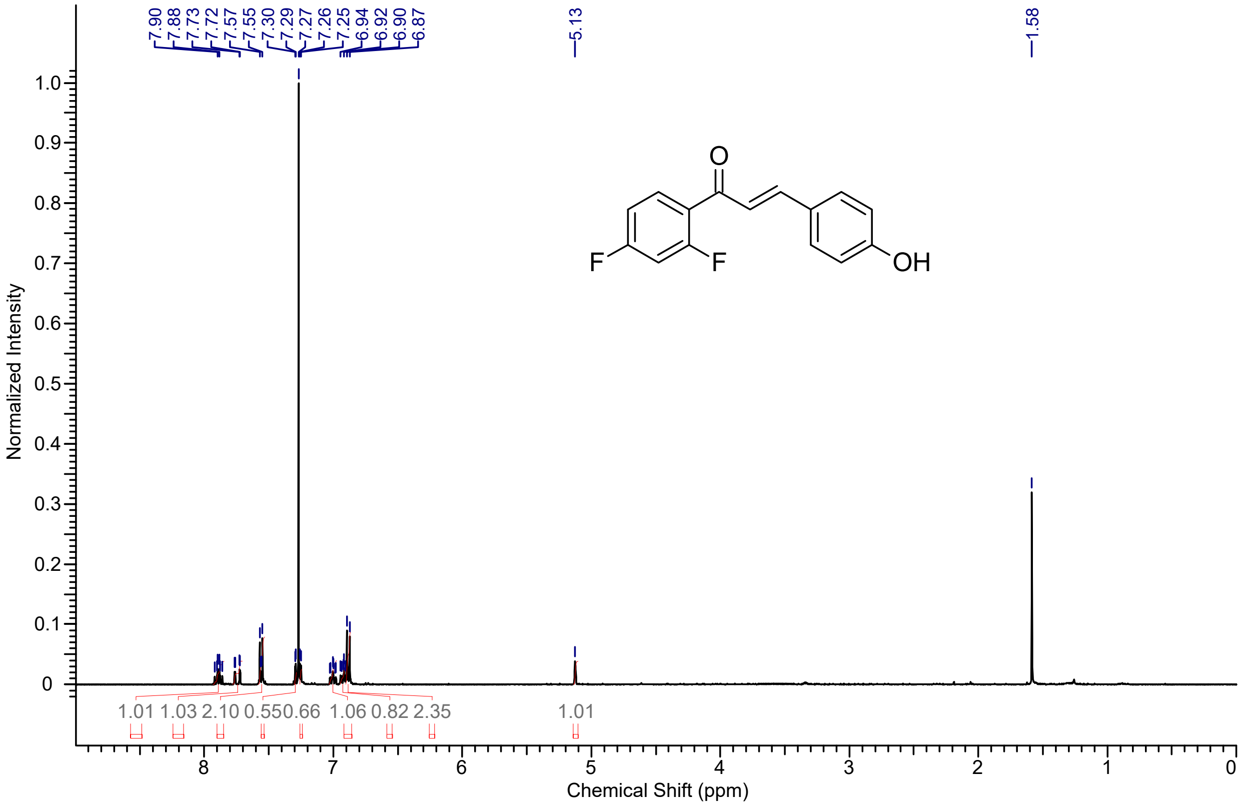

Figure S13b. ^{13}C NMR spectrum of the compound **13**

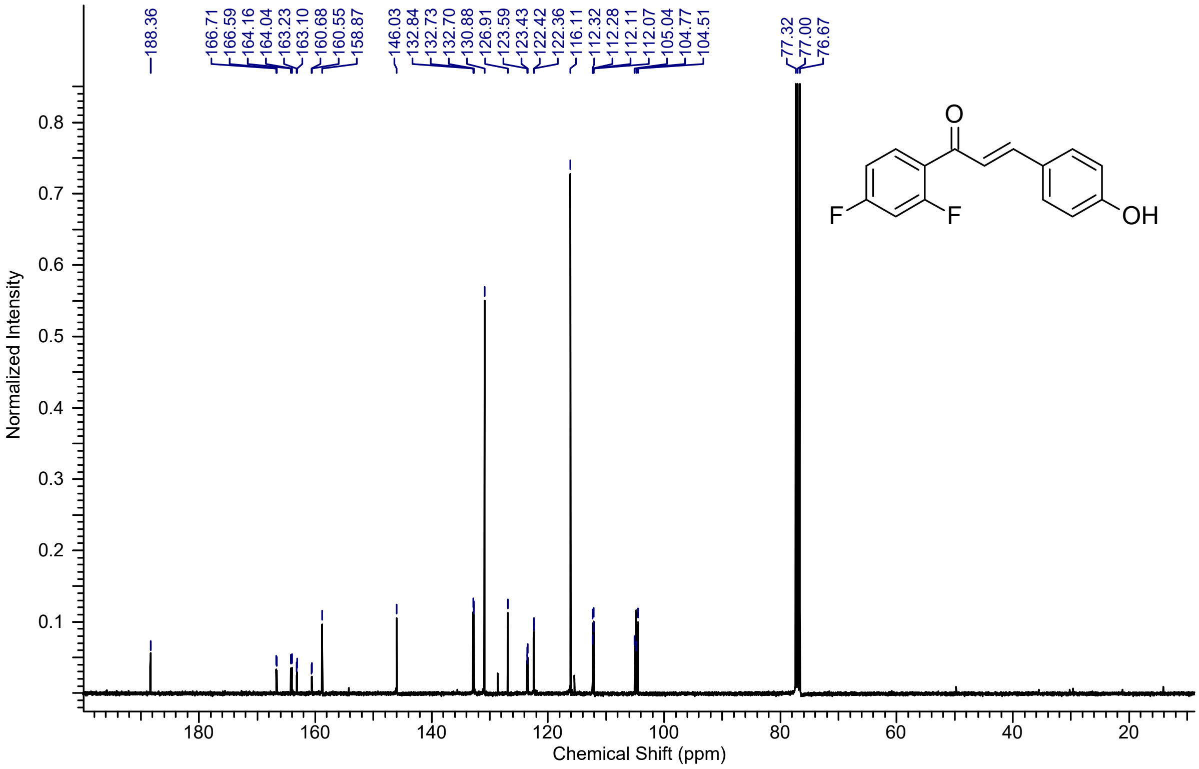

Figure S14. ILG and ILG derivatives suppress RANKL-induced osteoclast differentiation in RAW264.7 cells.

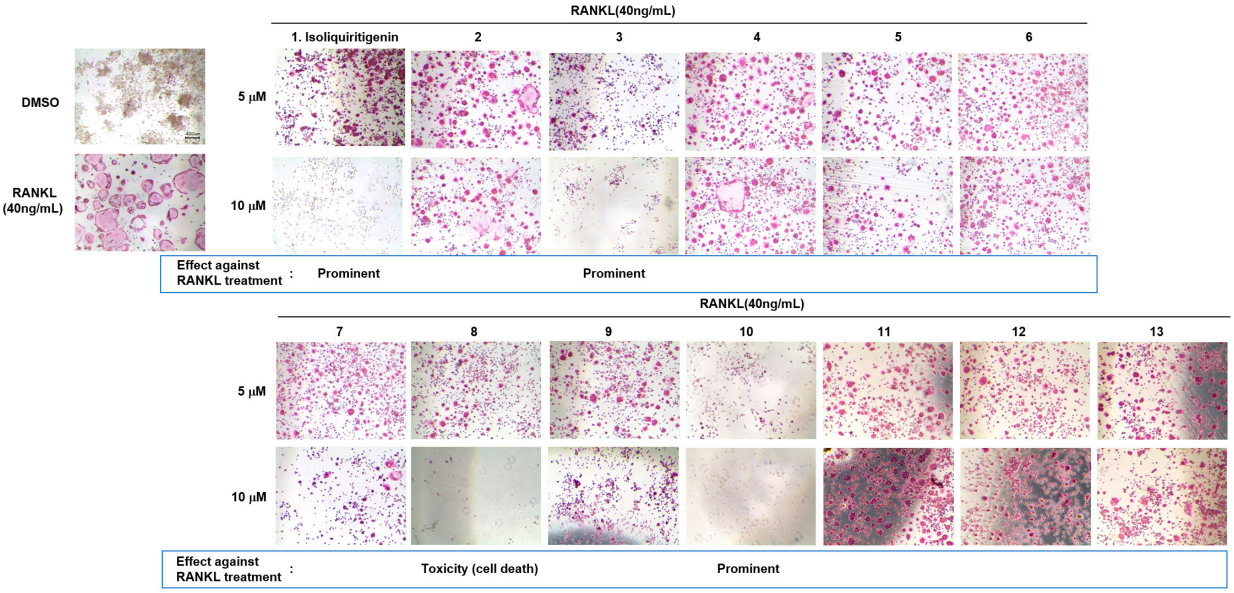

RAW264.7 cells were treated with 40 ng/ml RANKL in the presence of ILG or ILG compounds for 4 days. RAW264.7 cells were treated with 10 μM of ILG or ILG derivatives. The fixed cells were stained for TRAP and observed under a light microscope (40x) to determine TRAP positive cells. Scale bar = 400μm.

Table S1. Isoliquiritigenin and 12 derivatives.

Compound	structure	IUPAC name
Isoliquiritigenin (1)	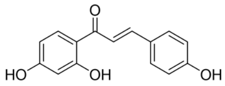	(*E*)-1-(2,4-Dihydroxyphenyl)-3-(4-hydroxyphenyl)prop-2-en-1-one
2	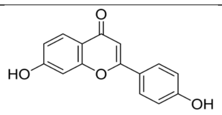	7-Hydroxy-2-(4-hydroxyphenyl)-4H-chromen-4-one
3	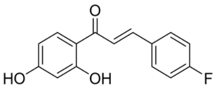	(*E*)-1-(2,4-Dihydroxyphenyl)-3-(4-fluorophenyl)prop-2-en-1-one
4	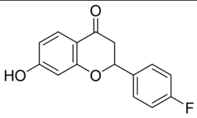	2-(4-Fluorophenyl)-7-hydroxychroman-4-one
5	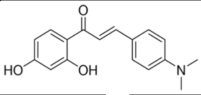	(*E*)-1-(2,4-Dihydroxyphenyl)-3-(4-(dimethylamino)phenyl)prop-2-en-1-one
6	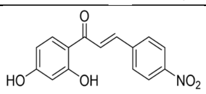	(*E*)-1-(2,4-Dihydroxyphenyl)-3-(4-nitrophenyl)prop-2-en-1-one

7	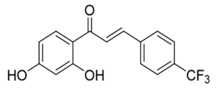	(*E*)-1-(2,4-Dihydroxyphenyl)-3-(4-(trifluoromethyl)phenyl)prop-2-en-1-one
8	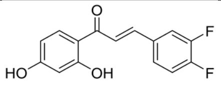	(*E*)-3-(3,4-Difluorophenyl)-1-(2,4-dihydroxyphenyl)prop-2-en-1-one
9	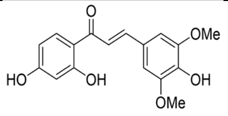	(*E*)-1-(2,4-Dihydroxyphenyl)-3-(4-hydroxy-3,5-dimethoxyphenyl)prop-2-en-1-one
10	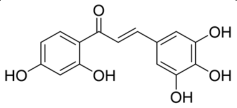	(*E*)-1-(2,4-Dihydroxyphenyl)-3-(3,4,5-trihydroxyphenyl)prop-2-en-1-one
11	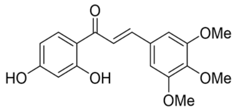	(*E*)-1-(2,4-Dihydroxyphenyl)-3-(3,4,5-trimethoxyphenyl)prop-2-en-1-one
12	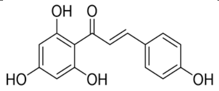	(*E*)-3-(4-Hydroxyphenyl)-1-(2,4,6-trihydroxyphenyl)prop-2-en-1-one
13	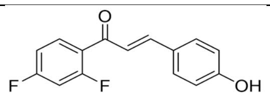	(*E*)-1-(2,4-Difluorophenyl)-3-(4-hydroxyphenyl)prop-2-en-1-one
